# Tensor-Driven Spectral CT Reconstruction via Noise-Adaptive Channel Weighting and Deterministic Block Matching

**DOI:** 10.1109/access.2026.3743930

**Published:** 2026-10-16

**Authors:** BAHAREH MOROVATI, CHANGSHENG FANG, SHUO HAN, LI ZHOU, SHUYI FAN, YU SHI, HENGYONG YU

**Affiliations:** Department of Electrical and Computer Engineering, University of Massachusetts Lowell, Lowell, MA 01854, USA

**Keywords:** Photon-counting computed tomography, variance-adaptive block sizing, enhanced channel weighting, tensor decomposition, sparse-view reconstruction

## Abstract

Photon-counting computed tomography (PCCT) enables revolutionary spectral imaging by capturing energy-resolved data in a single scan, offering unprecedented material discrimination and diagnostic capabilities. However, photon count limitations in individual energy channels result in severe noise amplification, degraded spatial resolution, and loss of fine anatomical details. Those challenges intensify in low-dose and sparse-view clinical protocols. To overcome these fundamental limitations, we propose a novel reconstruction framework that synergistically integrates three key innovations. First, we introduce an enhanced multi-metric channel weighting that automatically estimates signal-to-noise ratio per energy channel and dynamically prioritizes high-quality channels through multi-metric analysis by combining gradient magnitude, entropy, and spatial-spectral energy. Second, we employ deterministic hierarchical block clustering with Ward linkage for robust block matching, eliminating the initialization sensitivity of conventional k-means while improving grouping quality and improving the balance between detail preservation and noise suppression. These innovations are seamlessly integrated within a tensor decomposition framework that takes advantage of cross-channel correlations through low-rank factorization and enhanced sparsity constraints. Comprehensive validation on challenging extremity datasets acquired with a MARS PCCT scanner demonstrates substantial improvements in edge sharpness, noise suppression, and overall reconstruction quality compared with the conventional methods. Under 150 projection acquisition, the proposed framework achieves a SSIM of 0.9640±0.0198, PSNR of 36.35±0.0693 dB, and FSIM of 0.9698±0.1043 for Energy Channel 4. Our framework addresses critical reconstruction challenges in clinical PCCT, paving the way for robust multi-energy imaging.

## 1. INTRODUCTION

Conventional computed tomography (CT) has long served as the cornerstone of diagnostic imaging, offering fast and high-contrast visualization of anatomical structures [1]. However, it is fundamentally limited by spectral resolution and soft-tissue differentiation due to its reliance on energy-integrating detectors. The development of photon-counting detector (PCD) has greatly advanced CT imaging by enabling accurate energy discrimination and minimizing electronic noise. This breakthrough has positioned photon-counting CT (PCCT) as a premier imaging modality, particularly effective for multi-material decomposition in medical applications where precise spectral information is critical [2], [3], [4], [5], [6], [7], [8], [9]. In addition to improved material differentiation, PCCT enables higher spatial resolution and reduced beam-hardening artifacts. It also supports dose-efficient imaging by maintaining image quality at lower radiation exposure levels. Moreover, its ability to perform simultaneous multi-contrast imaging facilitates advanced clinical applications such as tissue characterization, plaque analysis, and functional imaging.

Unlike conventional detectors, PCDs count individual photons and record their energies, offering superior spatial resolution and better imaging performance. In PCCT imaging, the energy spectrum of the X-ray beam is divided into discrete channels, and the detected photon counts for each channel provide energy-specific attenuation information. This capability opens new possibilities in clinical diagnostics and research, such as virtual non-contrast imaging, plaque characterization, and tissue differentiation. Although PCD technology has advanced significantly, measurements still face degradation from low signal-to-noise ratios (SNR) caused by factors such as pulse pile-up, fluorescence effects, charge sharing, photon scattering, and other non-ideal detector behaviors [10]. These effects introduce non-linearity and distortion in the projection data, making it challenging to maintain the fidelity of reconstructed images. As a result, the imaging quality of individual PCCT energy channels is reduced, impacting intra-channel measurement accuracy, and compromising the precision of material decomposition. Addressing these limitations is critical not only to unlock the full potential of PCCT for high-quality spectral imaging, but also to ensure accurate quantitative assessments in clinical environments [6], [11], [12], [13].

Conventional reconstruction methods, such as filtered backprojection (FBP), are prone to degrade image quality due to the effects of quantum noise and photon count limitations, particularly in low-dose or sparse-view settings [14], [15]. Meanwhile, iterative reconstruction techniques, which integrate photon statistics and noise modeling, have been developed to enhance the quality of PCCT images by effectively suppressing noise and preserving fine details. These advanced approaches not only improve the signal-to-noise ratio but also allow flexible regularization strategies that incorporate prior knowledge or structural constraints [16], [17].

A notable framework, PRISM (Prior Rank, Intensity, and Sparsity Model), was designed to operate effectively with a reduced number of projections and lower radiation dose, making it suitable for dose-sensitive applications such as pediatric imaging [18]. Building on this, tensor-based methods have further investigated the spatial and spectral correlations between energy-channel images for improved multi-energy CT (MCT) reconstruction. For example, Semerci et al. proposed a tensor nuclear norm regularization algorithm, which was followed by the development of tensor dictionary learning (TDL) and its enhanced variant using *L*_0_-norm regularization (*L*_0_TDL), demonstrating significant advancements in noise suppression and structural preservation [19], [20], [21]. These methods are particularly effective in sparse-view imaging scenarios where data redundancy is limited and high-quality reconstruction is difficult to achieve with traditional techniques. However, despite these improvements, *L*_0_TDL still faces limitations that require further refinement [22], [23]. It effectively preserves fine structures and reduces artifacts, but relies on the availability of high-quality tensor dictionaries. This may not be feasible in practice and neglects spatial-spectral non-local similarity, limiting its adaptability to complex imaging tasks.

These challenges hinder its practical implementation in clinical workflows and motivate the search for more flexible and robust solutions. Deep learning and machine learning are delivering tangible clinical benefits in image quality, lesion detection, and early diagnosis, thereby improving workflow efficiency [24], [25], [26], [27], [28]. In recent years, deep learning approaches (*e.g.* convolutional neural networks (CNNs), generative adversarial networks (GANs), diffusion models, and transformer-based architectures) have shown remarkable promise in CT image reconstruction and noise reduction by leveraging large datasets to learn powerful representations of anatomical structures [6], [29], [30], [31], [32], [33], [34], [35], [36], [37], [38], [39]. Although these methods offer the state-of-the-art performance, they often require significant computational resources and extensive annotated training data, and can be sensitive to imaging variations such as acquisition protocols or scanner types. This raises concerns about their interpretability, robustness, and reliability in real-world medical applications, where consistency and trustworthiness are paramount. In some scenarios, incorporating specific parameter selection strategies can further improve model generalizability [40], [41], [42].

Based on Spectral-Image Similarity-based Tensor with Enhanced-Sparsity Reconstruction (SISTER) by Hu et al. [43], we propose a generalized framework that preserves SISTERs strength in exploiting spatial–spectral similarity via low-rank tensor decomposition while addressing its sensitivity to fixed patching size, its limited reliance on gradient-only channel weighting, and non-deterministic grouping. Specifically, we introduce three complementary innovations: (i) a *variance-adaptive block sizing* scheme that selects either 6 × 6 patches or 8 × 8 patches for the entire image according to its overall structural complexity, aligning the representation scale with global image complexity; (ii) an *enhanced multi-metric channel weighting with automatic noise estimation* that fuses gradient magnitude, entropy, and spatial–spectral energy with a robust, Median Absolute Deviation (MAD)-based per-channel SNR estimate, thereby prioritizing reliable energy channels without manual tuning; and (iii) *deterministic hierarchical block clustering* with Ward linkage to replace initialization-sensitive k-means, yielding reproducible, material-aware nonlocal groups for collaborative denoising. Integrated within an iterative reconstruction that couples a low-rank tensor prior with *ℓ*_0_ gradient sparsity, the resulting pipeline enhances noise suppression, preserves fine anatomical detail, and maintains spectral fidelity under sparse-view acquisitions. Comprehensive evaluations on preclinical PCCT datasets demonstrate consistent gains in PSNR, SSIM and FSIM, edge sharpness, and spectral accuracy, indicating strong potential for robust, sparse-view multi-energy imaging. Most of this work is based on the authors doctoral dissertation [44]. To clearly summarize the motivation, limitations, and contributions of this work, we provide the following structured overview:

- Motivation:
  Existing PCCT reconstruction methods still struggle to simultaneously achieve strong noise suppression, preserve fine anatomical structures, and stabilize spectral fidelity under sparse-view acquisition conditions.
- Key limitations of existing methods:
  - Conventional reconstruction methods such as FBP remain highly sensitive to sparse sampling and noise;
  - Tensor-based methods such as TDL and SISTER typically rely on availability of high-quality tensor dictionaries, fixed patch sizes and simplified channel weighting strategies, which limit adaptability to different image characteristics;
  - Clustering approaches such as k-means are sensitive to initialization and may lead to nonreproducible grouping results;
  - Deep learning methods often require large annotated datasets and substantial computational resources, while their robustness and interpretability may be limited in practical clinical settings.
- Aim and objectives:
  The aim of this work is to develop a robust and adaptive tensor-based reconstruction framework for sparse-view PCCT imaging. The main objectives are to improve channel weighting, enhance non-local similarity modeling, and ensure deterministic and reproducible reconstruction behavior.
- Novelty and main contributions:
  Building upon the SISTER framework [43], the proposed method introduces three key innovations:
  - variance-adaptive block sizing to better match overall image complexity;
  - enhanced multi-metric channel weighting with automatic noise estimation to adaptively prioritize reliable spectral channels;
  - deterministic hierarchical block clustering to replace initialization-sensitive grouping methods and improve reproducibility.
  Together, these improvements enhance reconstruction quality, robustness, and consistency under sparse-view conditions.

Table 1 gives a list of the acronyms and abbreviations used in this paper.

The rest of this paper is structured as follows. Section II introduces the proposed framework, Section III outlines the experimental setup and results, and Section IV discusses the method’s benefits for noise suppression and detail preservation, concluding the study.

## II. METHODS

### A. OVERVIEW

Figure. 1 summarizes our reconstruction pipeline for PCCT image reconstruction, designed to recover diagnostic-quality images from sparse, noisy, energy-resolved measurements. The diagram presents the sequential processing steps of the reconstruction pipeline, starting from initialization of the image tensor and followed by iterative reconstruction and prior-guided refinement. The method augments a tensor-based prior with three synergistic components:

1. **Variance-adaptive block sizing.** We automatically select a single patch (block) size for each reconstructed image based on its global variance. Images with higher global variance use 6 × 6 blocks, whereas images with lower global variance use 8 × 8 blocks, thereby balancing detail preservation and noise suppression according to the overall structural complexity of the image.
2. **Enhanced Multi-Metric Channel Weighting with Automatic Noise Estimation.** A multi-metric quality score (gradient magnitude, entropy, spatial–spectral energy) is combined with automatic per-channel SNR estimation to prioritize reliable energy channels to mitigate photon starvation in low-energy channels while leveraging the stronger statistics of higher-energy channels.
3. **Deterministic hierarchical block clustering.** It replaces initialization-sensitive k-means with ward-linkage agglomerative clustering, yielding reproducible, clinically reliable nonlocal block groups for subsequent collaborative denoising.

These modules are seamlessly integrated into an iterative optimization framework to exploit cross-channel correlations through low-rank tensor decomposition while preserving edges via *l*_0_-gradient sparsity. During each iteration, the current image tensor is first updated through the OS-SART-based data-consistency (DC) step to enforce consistency with the measured projection data. The updated tensor is then processed using the proposed enhanced multi-metric channel weighting, variance-adaptive block extraction, and deterministic hierarchical clustering. CP tensor decomposition is subsequently performed on each block group, where the factor matrices (U) and tensor coefficients (T) are alternately updated using the alternating least squares (ALS) algorithm. Finally, the reconstructed spectral image tensor (X) is obtained through block aggregation before the next iteration begins. This iterative procedure progressively refines the reconstructed spectral image tensor, improving noise suppression while maintaining spectral fidelity and preserving fine anatomical details.

### B. TENSOR DECOMPOSITION FOR PCCT RECONSTRUCTION

Tensor decomposition is the cornerstone of the SISTER framework and forms the mathematical basis for representing high-dimensional PCCT data. In PCCT, multi-energy projections in energy channels create a natural structure for representing the data as a third-order tensor. Each element in this tensor encodes spatial and spectral information, making it ideal for modeling correlations in energy channels and spatial dimensions [45], [46]. By decomposing tensors into weighted rank-one components, Candecomp/Parafac (CP) decomposition captures spatial-spectral correlations, aiding in noise suppression and structural preservation. Unlike fixed dictionary methods, CP dynamically adapts to the data, ensuring accurate representation of complex structures. This approach preserves fine details and edges, especially in low-dose and sparse-view scenarios, forming a robust foundation for adaptive spatial clustering and channel-specific weighting in our proposed enhancements [43]. A tensor can be viewed as a multidimensional data structure. An N-order tensor is represented as $X\in R^{I_{1}\times I_{2}\times \cdots \times I_{N}}$, where each entry is denoted by $x_{i_{1}i_{2}\cdots i_{N}}$ with $1\leq i_{n}\leq I_{N}$ for n=1,…,N. For the special cases N=1 and N=2, the tensor corresponds to a vector and a matrix, respectively. Generally speaking, CP decomposition breaks down an $N^{\mathrm{th}}$-order tensor x into a weighted combination of rank-one tensors

(1) $$X=\sum_{h=1}^{H}\lambda_{h}a_{1h}\circ a_{2h}\circ \cdots \circ a_{Nh}\;\;=[[\lambda ;A_{1},A_{2},\ldots ,A_{N}]],$$

where $\lambda_{h}$ represents the weighting function associated with each rank-one component of the tensor decomposition, $a_{nh}\in R^{I_{n}},(n=1,\ldots ,N)$ is a normalized vector, the symbol ″∘″ denotes the outer product, $A_{n}$ refers to factor matrices, $\lambda =\left[\lambda_{1},\lambda_{2},\ldots ,\lambda_{H}\right]\in R^{H}$, and the factor matrix for mode n is $A_{n}=\left[a_{n1},a_{n2},\ldots ,a_{nH}\right]\in R^{I_{n}\times H}$. The sparsity level of the representation can be managed by tuning the parameter *H* [47]. CP decomposition dynamically adapts to the underlying data, capturing the spatial-spectral correlations that are critical for preserving tissue structures and suppressing noise in PCCT reconstructions. For PCCT, where photon count in each energy channel is low, CP decomposition provides an optimal representation of the sparse and noisy data. It models relationships between energy channels, enabling better differentiation of materials in spectral CT imaging. Unlike traditional methods, CP decomposition learns directly from the data, allowing it to adapt to complex structures unique to PCCT. This makes it particularly effective for handling challenges like sparse-view imaging and low-dose scenarios.

### C. VARIANCE-ADAPTIVE BLOCK SIZING

In tensor-based spectral CT reconstruction, the choice of patch (block) size for dictionary learning significantly impacts the trade-off between detail preservation and noise suppression. Fixed block sizes, as employed in conventional approaches such as in SISTER [43], fail to account for the spatially-varying complexity inherent in medical images. To address this limitation, we introduce a variance-adaptive block sizing strategy that automatically selects optimal block dimensions based on global image characteristics as illustrated in Fig. 2.

Our approach computes the global variance of the normalized multi-channel image $X\in R^{I\times J\times S}$ as:

(2) $$\sigma^{2}=\frac{1}{IJS}\sum_{i=1}^{I}\sum_{j=1}^{J}\sum_{s=1}^{S}{\left(X_{i,j,s}-\mu \right)}^{2},$$

where *I*, *J*, and *S* denote image height, width, and number of spectral channels, respectively, and *μ* is the global mean intensity. The standard deviation *σ* serves as a scalar complexity measure, characterizing the overall image content.

Based on this variance estimate, we apply an adaptive block size selection rule:

(3) $$b_{\text{size}}=\left\{\begin{array}{ll} 6\times 6, & \text{if}\;\sigma >\tau \\ 8\times 8, & \text{if}\;\sigma \leq \tau \end{array}\right.$$

Where *τ* denotes the variance threshold to determine the adaptive block size. In this study, *τ* = 0.20 is selected based on a sensitivity analysis performed on the MARS PCCT dataset. Several representative threshold values are evaluated, and *τ* = 0.20 provides the best overall reconstruction performance by achieving the most favorable balance between detail preservation and noise suppression. The block-size decision is performed once for each reconstructed multi-channel image based on its global variance. Consequently, all extracted blocks use the same block size (either 6 × 6 or 8 × 8), Images with *σ* > 0.20 typically contain significant fine structures, edges, and texture, requiring smaller blocks (6 × 6) for adequate detail capture. *σ* ≤ 0.20 indicates relatively homogeneous contents where standard blocks (8 × 8) are sufficient.

The smaller 6 × 6 blocks (*b*_size_ = 36 pixels) provide finer spatial resolution in the dictionary learning process, enabling a better representation of high-frequency anatomical features. When smaller blocks are selected, they reduce the likelihood of mixing dissimilar patterns, improving cluster homogeneity. The extracted blocks $B\in R^{b_{\text{size}}\times b_{\text{size}}\times S\times N_{\text{blocks}}}$ are subsequently processed through clustering and CP tensor decomposition.

To ensure dimensional consistency throughout the reconstruction pipeline, we verify that the reconstructed image $\tilde{X}$ from joint block aggregation matches the original dimensions. If size mismatch occurs (due to different block tiling), we apply bilinear interpolation:

(4) $${\tilde{X}}_{\text{corrected}}=resize(\tilde{X},[I,J]).$$

This automatic correction guarantees seamless integration with the iterative optimization framework without introducing size-related errors. The adaptive block sizing strategy thus provides a simple, data-driven mechanism to balance detail preservation and noise suppression, adapting automatically to the overall structural complexity of the image without manual tuning [48].

### D DETERMINISTIC HIERARCHICAL BLOCK CLUSTERING

Block clustering is a critical component in tensor-based spectral CT reconstruction, as it groups similar image patches before applying low-rank tensor decomposition for collaborative denoising. Traditional approaches employ k-means clustering due to its computational efficiency [43]. However, k-means suffers from two fundamental limitations: sensitivity to random initialization leading to non-reproducible results, and tendency to converge to suboptimal local minima [49], [50]. These issues are particularly problematic for quantitative medical imaging where identical input data must yield identical outputs for reliable measurements and longitudinal studies.

We address these limitations by replacing k-means with hierarchical agglomerative clustering using Ward’s minimum variance linkage criterion [51]. Given N extracted image blocks represented as feature vectors $x_{1},x_{2},\ldots ,x_{n}\in R^{d}$, where *d* = block_size^2^ × n_channels, we first compute the pairwise Euclidean distance matrix D. Ward’s linkage then builds a hierarchical tree by iteratively merging clusters that produce the minimum increase in total within-cluster variance [52], following the criterion:

(5) $$\Delta \left(C_{i},C_{j}\right)=\frac{\left|C_{i}\right|\left|C_{j}\right|}{\left|C_{i}\right|+\left|C_{j}\right|}{\left\|v_{i}-v_{j}\right\|}^{2},$$

where *C_i_* and *C_j_* are clusters with centroids ***v****_i_* and ***v****_j_*. The resulting dendrogram is subsequently cut to obtain the desired number of clusters, *K*. Because hierarchical clustering exhibits quadratic computational complexity with respect to the number of image blocks, directly clustering all *N* blocks becomes computationally prohibitive for large-scale datasets. To improve computational efficiency, a hybrid clustering strategy is adopted when *N* > 3, 000. Specifically, hierarchical clustering is first performed on a representative subset of *M* = 3, 000 randomly sampled blocks. A fixed random seed is employed during the sampling process to ensure that identical input data always produce the same sampled subset across repeated runs, thereby preserving the reproducibility of the overall clustering procedure. The resulting cluster centroids are then used to assign the remaining blocks according to the nearest-centroid criterion. Consequently, the computational complexity is reduced from approximately $𝒪(N^{2})$ to $𝒪(M^{2}\;+\;\mathrm{NK})$, where *M* = 3, 000. For the reconstructed 512 × 512 images in this study (8 × 8 patches). The sampling size is empirically selected to balance clustering quality and computational efficiency, providing representative centroid estimation while substantially reducing the overall computational cost.

This hierarchical approach offers three key advantages over k-means. First, it is completely deterministic, given the same distance matrix, it always produces identical cluster assignments, eliminating the run-to-run variability inherent in k-means. Second, by building a global hierarchical view before partitioning, it avoids local minima that trap k-means, yielding superior cluster cohesion and separation. Third, it naturally discovers material-specific groupings in spectral data, as blocks with similar energy-dependent attenuation patterns progressively merge based on their spectral signatures rather than arbitrary geometric proximity. These improved clusters lead to better CP tensor decomposition because grouped blocks share truly similar spectral characteristics, enabling extraction of more representative low-rank components. Quantitative evaluation demonstrates that hierarchical clustering contributes approximately 0.3–0.8 dB PSNR improvement over k-means while simultaneously ensuring perfect reproducibility, a dual benefit critical for clinical translation of spectral CT reconstruction methods.

### E ENHANCED MULTI-METRIC CHANNEL WEIGHTING WITH AUTOMATIC NOISE ESTIMATION

PCCT generates multiple energy channels with inherently different signal quality characteristics. Low-energy channels suffer from elevated noise due to photoelectric absorption and beam hardening, while high-energy channels benefit from better penetration but exhibit reduced contrast. These varying noise levels and information content necessitate intelligent channel prioritization rather than uniform weighting. We propose an enhanced channel weighting strategy that combines multi-metric quality assessment with automatic noise estimation to adaptively prioritize reliable spectral channels.

For each energy channel *s*, we compute three complementary quality metrics. Before computing the quality metrics, each energy channel is first intensity-normalized to the range [0, 1] to ensure that the subsequent quality measures are evaluated on a consistent intensity scale. The gradient magnitude $G_{S}$ captures edge strength and structural information through $G_{s}=\sum \left|\nabla X_{s}\right|$, where $\nabla X_{s}$ represents the spatial gradient. The Shannon entropy $M_{s}$ quantifies textural complexity and information content as $M_{s}=-\sum p(i)logp(i)$, where p(i) is the normalized histogram. The spatial-spectral energy ratio $E_{s}=\frac{\sum \left|\nabla X_{s}\right|}{\sum X_{s}^{2}}$ balances structural content against overall intensity contribution, where $X_{s}$ denotes the reconstructed image corresponding to the $s^{\mathrm{th}}$ energy channel. These metrics are combined as:

(6) $$Q_{s}=\alpha \cdot G_{s}+\beta \cdot M_{s}+\gamma \cdot E_{s},$$

with weights *α* = 0.5, *β* = 0.3, and *γ* = 0.2 empirically optimized to prioritize structural information.

Critically, we extend this quality-based weighting with automatic per-channel noise estimation using the robust Median Absolute Deviation (MAD) method. The noise standard deviation $\sigma_{\text{noise},s}$ is estimated from high-frequency components via the Laplacian operator $\nabla^{2}X_{s}$:

(7) $$\sigma_{\text{noise},s}=1.4826\cdot median\left(\left|\nabla^{2}X_{s}-median\left(\nabla^{2}X_{s}\right)\right|\right).$$

We scale the MAD by 1/Φ^−1^(3/4) ≈ 1.4826 to obtain a Fisher-consistent estimator of the Gaussian standard deviation [53]. From this, we compute the signal-to-noise ratio ${SNR}_{s}=\mu_{s}/\sigma_{\text{noise},s}$, where $\mu_{s}$ is the mean channel intensity. The final adaptive weight incorporates both quality metrics and SNR:

(8) $$w_{s}=Q_{s}\cdot \left(0.8+0.2\cdot {SNR}_{s}^{\text{norm}}\right),$$

where ${SNR}_{s}^{\text{norm}}$ denotes the normalized SNR across channels. The SNR modulation factor is intentionally limited to a maximum adjustment of 20%, modestly favoring high-SNR channels while preserving the primary contribution of the structure-based quality metrics and preventing excessive weighting differences across spectral channels.

This noise-aware weighting strategy offers three advantages over uniform channel treatment. First, it automatically identifies and emphasizes channels with superior signal quality without manual parameter tuning, critical for clinical workflows. Second, the MAD-based noise estimator is robust to outliers and does not require noise-free reference images, making it practical for real data. Third, by combining structural quality metrics (*G*, *M*, *E*) with statistical reliability (SNR), the framework ensures that channels contribute proportionally to both their information content and measurement confidence. Experimental validation demonstrates that enhanced weighting reduces noise propagation from low-quality channels while preserving structural consistency across spectral channels. This automatic, data-driven weighting strategy is particularly effective in sparse-view scenarios, where reliable utilization of multi-channel information is essential for robust spectral CT reconstruction.

### F. INTEGRATION WITH SPARSITY-DRIVEN RECONSTRUCTION

The proposed framework integrates variance-adaptive block sizing, deterministic hierarchical block clustering, and enhanced channel weighting with tensor decomposition and sparsity-based reconstruction to enhance denoising and feature recovery. The mathematical model can be formulated as:

(9) $$\underset{X,{\left\{{\overset{ˆ}{Z}}_{k}\right\}}_{k=1}^{K}}{min}\frac{1}{2}\sum_{s=1}^{S}{\left\|AX_{s}-y_{s}\right\|}_{2}^{2}+\frac{\mu }{2}\sum_{s=1}^{S}{\left\|\nabla X_{s}\right\|}_{0}+\frac{\lambda }{2}\sum_{k=1}^{K}{\left\|Z_{k}(X)-{\overset{ˆ}{Z}}_{k}\right\|}_{F}^{2},\;\text{s.t.}{\overset{ˆ}{Z}}_{k}=\left[\lambda_{k};A_{1},A_{2},A_{3},A_{4}\right],\;k=1,\ldots ,K,$$

where *X* is a 3*^rd^*-order reconstructed image tensor, *A* represents the CT system matrix, *Z_k_* (*X*) denotes clustered tensor patches, λ and *μ* are regularization parameters. *S* represents the number of spectral (energy) channels in PCCT, *k* denotes the index of clusters used in tensor decomposition and *y_s_* represents the measured data for the *S^th^* energy channel. The data are collected by the detector during the CT scan. ${\overset{ˆ}{Z}}_{k}$ represents the learned or approximated tensor decomposition coefficients for the *k^th^* cluster. It is used as the target or reference during the reconstruction process, derived from tensor decomposition techniques. The regularization parameters λ and *μ* are adopted from the original SISTER framework [43], which employs the same CP tensor decomposition and *ℓ*_0_-gradient sparsity formulation. Because the proposed method preserves the same optimization model while introducing improvements in channel weighting, adaptive block sizing, and deterministic hierarchical clustering, the empirically optimized parameters in [43] are retained throughout all experiments. The optimization alternates between solving and updating tensor coefficients using methods such as Alternating Least Squares (ALS) [43]. ALS has demonstrated robust practical performance for tensor factorization problems. Furthermore, the proposed adaptive block-size selection and hybrid hierarchical clustering improve computational efficiency and facilitate scalability to larger datasets. This formulation captures multi-dimensional correlations in PCCT data while incorporating spatial-spectral clustering and adaptive channel weights. This combination not only extends the foundational principles of SISTER but also significantly improves its robustness and efficacy in sparse-view imaging applications, achieving superior noise suppression, edge preservation, and spectral fidelity.

## III. EXPERIMENTS AND RESULTS

### A. DATASET

To evaluate the effectiveness of the proposed framework, we use clinical extremity scans acquired on a high-resolution MARS PCCT scanner (MARS Bioimaging Ltd, Christchurch, New Zealand). The dataset consists of patient wrist imaging results, containing 888 image slices, The MARS scanner employs a multi-chip detector array composed of 12 chips arranged in 3 quads, with each quad comprising 4 chips positioned at fixed angles to form an arc, referred to as the “camera”. Each chip contains a 128 × 128 grid of square pixels, with a pixel size and spacing of 0.11 mm. During acquisition, 373 views per rotation are captured, covering energy thresholds from 7 keV to 70 keV across 5 spectral channels. The source-to-detector distance is set to 271.88 mm, and the source-to-object distance is 199.52 mm, enabling high-resolution imaging of fine anatomical structures. For each view and energy channel, photon counts are recorded, providing rich spectral data.

The resulting reconstructed PCCT volumes have an in-plane resolution of 512×512 pixels per slice, with 5 spectral channels. Prior to reconstruction, the raw helical cone-beam projection data are preprocessed and rebinned into equiangular fan-beam geometry to facilitate compatibility with our reconstruction framework [54]. For quantitative evaluation, the reference images used for SSIM, PSNR and FSIM computation are reconstructed using the SART algorithm with the full 373 projections views and 200 iterations. These full-view reconstructions serve as high-quality references to evaluate sparse-view reconstruction methods. Since true ground truth images are generally unavailable in real PCCT acquisitions, such full-view reconstructions are commonly used as practical reference images. However, it should be noted that the reference images are still affected by reconstruction noise and algorithm-dependent bias and therefore represent approximate rather than absolute ground truths. Throughout this paper, the term “ground truth (GT)” or “reference image” refers to this high-quality reference reconstruction rather than an actual ground truth image. The dataset characteristics and evaluation protocol are summarized in Table 2. This research study was conducted retrospectively using human subject data. Ethical approval was not required as confirmed by the Ethics Committee of UMass Lowell (04/25/2023, No.23-054).

### B. EXPERIMENTAL SETTINGS AND EVALUATION METRICS

The experiments are conducted on an Ubuntu 22.04. LTS system featuring an Intel(R) Core(TM) i9–9920X CPU @ 3.50GHz. All models are implemented using MATLAB 2023 utilizing two NVIDIA RTX 6000 Ada GPUs to accelerate computations.

To assess the effectiveness of the proposed approach, we use two widely adopted image quality metrics: Structural Similarity Index Measure (SSIM), Peak Signal-to-Noise Ratio (PSNR) and FSIM. SSIM evaluates the perceptual similarity between reconstructed and reference images by analyzing luminance, contrast, and structural components, with values closer to 1 indicating higher visual fidelity. To enhance interpretability, SSIM maps are also generated to visualize localized structural differences across the image, where brighter regions correspond to areas of higher similarity and darker regions highlight local inconsistencies. PSNR, computed in the reconstructed image domain, quantifies the ratio of signal strength to noise, with higher values signifying better image quality and lower distortion. In addition, we incorporate the Feature Similarity Index Measure (FSIM), which evaluates image quality based on phase congruency and gradient magnitude, providing a complementary assessment of perceptual quality and structural fidelity, particularly in preserving fine image details. Additionally, power spectra are analyzed to characterize the frequency content of the reconstructed images. By comparing the spectral distribution across methods, we assess how well each approach preserves frequency details while suppressing noise, providing complementary insight beyond spatial-domain metrics. Since the proposed method is an iterative optimization-based reconstruction framework rather than a data-driven learning model, each dataset is independently reconstructed from the corresponding sparse-view projection data without any prior training phase. For evaluation, the reconstructed images are quantitatively compared against reference images (full-view reconstructions) using PSNR, SSIM and FSIM metrics. The reported results represent averages across multiple slices to ensure robustness and avoid bias toward individual examples. All PSNR, SSIM and FSIM values reported in the tables are averaged across all reconstructed slices, and the corresponding standard deviations are included to indicate variability across slices.

### C. RESULTS AND COMPARISON ANALYSIS

Comprehensive comparisons are conducted against several the state-of-the-art reconstruction methods, including filtered backprojection (FBP) as the baseline analytical approach [55], simultaneous algebraic reconstruction technique (SART) as an iterative reconstruction approach [56], TDL [47], SISTER [43], and learned Tensor Neural Network (TNN) texture prior [14], a deep learning-based method leveraging tensor-based priors for enhanced texture and noise suppression.

For evaluation, experiments are performed under sparse-view scenarios, and 150 views and 100 views are extracted from the 373 projections. For the case of 150 projections, our framework demonstrates clear advantages in both noise suppression and structure preservation, as shown in Fig. 3. Without advanced priors, FBP reconstructions exhibit pronounced noise and streak artifacts, with substantial loss of fine detail and structural clarity. SART lowers the noise floor but leaves residual artifacts and noticeable blurring. TDL further improves image quality, yet residual noise and artifacts remain. SISTER achieves stronger noise suppression and better structure preservation, though limitations in edge sharpness and spectral fidelity persist. TNN, leveraging tensor priors, enhances texture retention and denoising but can introduce artifacts in homogeneous regions. In contrast, our framework consistently preserves textures and edges while reducing noise, yielding higher overall image quality than competing methods. The second and fourth rows in Fig. 3 present the SSIM maps between each methods output and the ground truth, highlighting spatial similarity at a local level. Brighter regions in the SSIM maps indicate higher similarity, further confirming the structural fidelity of our results. Quantitative evaluations using SSIM and PSNR metrics are summarized in Table 3.

For the case of 100 projection views, results are visualized in Fig. 4. This setting poses significant challenges due to insufficient angular sampling. Despite this, our framework maintains superior image quality, delivering sharper edges, reduced artifacts, and more accurate structural recovery compared to competing approaches. While the TNN preserves textural information, it struggles to eliminate streak artifacts and noise in highly sparse regions. The SSIM maps in the second and fourth rows of Fig. 4 clearly illustrate this improvement, where our method achieves significantly higher local similarity across the entire image. Our method consistently yields the highest SSIM, PSNR and FSIM values in different energy channels, as shown in Table 4, underscoring its robustness in data-limited scenarios. Moreover, we analyzed the power spectra for the case of 150 projection views.

The power spectra, shown in Fig. 5, illustrates the frequency characteristics of the reconstructed images under sparse-view (150 projections) scenario. Low-frequency components correspond to the overall image structure, whereas high-frequency components are associated with fine details as well as noise. As observed in Fig. 5, FBP, SART, and TNN preserve similar low-frequency structures but exhibit higher power in the high-frequency region, indicating increased noise. In contrast, the proposed method produces a power spectrum that most closely follows the ground truth curve across a broad frequency range (approximately 0.1–0.7 normalized frequency). This indicates that the proposed approach effectively suppresses noise while better preserving the underlying frequency characteristics of the ground truth image compared with FBP, SART, SISTER, TDL, and TNN.

Overall, these results highlight the strength of our framework in adapting to different data fidelity conditions. It not only improves reconstruction accuracy but also ensures spectral consistency and robustness, particularly in PCCT applications when traditional methods fail.

## D. PARAMETER SENSITIVITY ANALYSIS

### 1) SENSITIVITY ANALYSIS OF THE VARIANCE THRESHOLD (*τ*)

To evaluate the influence of the variance threshold (*τ*) in the proposed adaptive block sizing strategy, a sensitivity analysis is conducted using five representative threshold values (*τ* = 0.10, 0.15, 0.20, 0.25, and 0.30). The quantitative results are summarized in Table 5. Among the evaluated values, *τ* = 0.20 achieves the highest SSIM, PSNR, and FSIM, indicating the best trade-off between preserving fine structural details and suppressing image noise. Lower threshold values tend to classify more regions as high variance, resulting in smaller block sizes and reduced denoising efficiency. In contrast, greater threshold values favor larger blocks, leading to increased smoothing and a slight loss of structural details. Overall, the proposed framework remains relatively stable over a reasonable range of threshold values, while *τ* = 0.20 consistently provides the best reconstruction performance on the MARS PCCT dataset.

### 2) SENSITIVITY ANALYSIS OF THE WEIGHTING COEFFICIENTS *α*, *β* AND *γ*

To justify the selected weighting coefficients, a sensitivity analysis is conducted using several representative weight combinations satisfying *α* + *β* + *γ* = 1. The quantitative results are summarized in Table 6. Among the evaluated combinations, the selected weights (*α* = 0.5, *β* = 0.3, and *γ* = 0.2) consistently achieves the best reconstruction performance on the MARS PCCT dataset in terms of SSIM, PSNR, and FSIM. Although fixed weighting coefficients are adopted in the proposed framework, learning channel-adaptive weights directly from the data may further improve reconstruction performance and will be investigated in future work.

### E. ABLATION STUDY

To evaluate the contribution of each proposed component, we conduct an ablation study in which modules are added stepwise to a common tensor-based baseline. The ablation study is performed using 100 projection dataset because it represents the most challenging sparse-view scenario, where the contribution of each proposed module is most evident. Since the proposed framework consistently improves reconstruction quality under both the 100 and 150 projection settings, only 100 projection ablation results are presented for clarity and conciseness. Future work will investigate the contribution of each module under additional sparse-view and dose conditions. All experiments use the same MARS PCCT extremity dataset under sparse-view acquisition (100 projections). The following configurations summarize the incremental steps used to demonstrate the effectiveness of each component:

1. **Tensor-only (SISTER-equivalent)**: tensor decomposition with standard k-means, *gradient-only* channel weighting, and fixed 8 × 8 patches.
2. **Channel Weighting**: add our SNR-aware *multi-metric* channel weighting to the first step; retain k-means and fixed patches.
3. **Hierarchical Clustering**: replace k-means with deterministic Ward-linkage clustering in the first step; use *gradient-only* channel weighting and fixed patches.
4. **Adaptive Blocks**: use variance-adaptive 6 × 6/8 × 8 patches in the first step; retain k-means and gradient-only channel weighting.
5. **Full Method**: integrate all three components (channel weighting, hierarchical clustering, adaptive blocks).

Table 7 reports metrics for the 1*^st^* and 4*^th^* energy channels. Introducing enhanced multi-metric channel weighting with automatic noise estimation on top of *Tensor-only* provides a consistent improvement by prioritizing reliable channels. Deterministic hierarchical clustering contributes a further ~ 0.3 dB over *Tensor-only* and improves reproducibility. Variance adaptive block sizing adds ~ 0.3 dB with edge benefits. The *Full Method* reaches 34.42 dB and 34.29 dB PSNR for 1*^st^* and 4*^th^* energy channels, respectively. Fig. 6 (4*^th^* energy channel) shows that *Tensor-only* suppresses noise but slightly over-smooths fine structure; adding enhanced multi-metric channel weighting with automatic noise estimation cleans homogeneous regions and improves spectral consistency; hierarchical clustering sharpens boundaries and textures; variance adaptive block sizing better preserve fine trabecular patterns; the *Full Method* combines these benefits with minimal artifacts.

### F. COMPUTATIONAL COST

Unlike deep learning methods, the proposed framework is an optimization-based reconstruction algorithm and therefore does not require a training stage. Its computational cost is primarily determined by the iterative reconstruction process, tensor decomposition, and block clustering. Specifically, each outer iteration consists of the following major computational stages: (i) OS-SART reconstruction, (ii) enhanced multi-metric channel weighting, (iii) variance-adaptive block extraction, (iv) deterministic hierarchical clustering, (v) CP tensor decomposition, and (vi) block aggregation.

Among these components, CP tensor decomposition constitutes the dominant computational cost, while deterministic hierarchical clustering introduces additional overhead. To improve scalability, a hybrid clustering strategy is employed when the number of extracted image blocks exceeds 3,000. Instead of performing hierarchical clustering on all image blocks, clustering is first carried out on a representative subset of *M* = 3, 000 randomly sampled blocks. The resulting cluster centroids are then used to assign the remaining blocks according to the nearest-centroid criterion. Consequently, the clustering complexity is reduced from approximately *O*(*N*^2^) to *O*(*M*^2^+*NK*), where *N* denotes the total number of image blocks and *K* is the number of clusters.

For the reconstructed 512 × 512 images in this study, the proposed hybrid strategy reduces the number of pairwise distance evaluations from approximately 2.05 × 10^9^ to 4.5 × 10^6^ , corresponding to nearly a 450× reduction. Including the nearest-centroid assignment step, the overall clustering cost remains approximately 80× lower than that of full hierarchical clustering.

Table 8 summarizes the computational cost and memory usage of all reproduced optimization-based reconstruction methods under the same experimental environment. The proposed method requires 140 minutes for complete reconstruction, compared with 168.0 minutes for SISTER, corresponding to a 17% reduction in runtime. This improvement is achieved despite the introduction of adaptive block sizing and deterministic hierarchical clustering, which are compensated by the proposed hybrid clustering strategy that substantially reduces the computational complexity of block grouping. As a result, the additional processing introduced by the proposed framework has only a minimal impact on the overall computational burden while providing significant improvements in reconstruction quality.

Compared with TDL and TNN, the proposed framework requires longer reconstruction time because of the additional iterative CP tensor decomposition and tensor optimization procedures. These additional computations, however, enable superior exploitation of the low-rank structure and spectral correlations among energy channels, leading to improved reconstruction accuracy and structural preservation. In terms of memory consumption, the proposed method requires 654 MB, which is only slightly higher than SISTER (643 MB) and remains practical for modern computing platforms.

Since CP tensor decomposition is independently performed for each block group, the proposed framework is naturally suitable for parallel implementation, providing considerable potential for further acceleration on multi-core CPUs and GPU architectures.

## IV. DISCUSSION AND CONCLUSION

The proposed framework introduces three complementary enhancements that improve tensor-based spectral CT reconstruction. First, the proposed enhanced multi-metric channel weighting with automatic noise estimation improves conventional gradient-magnitude-based channel weighting by incorporating complementary image quality metrics for more robust channel prioritization. By combining automatic SNR estimation via robust median absolute deviation with multi-metric quality assessment (gradient magnitude, entropy, spatial-spectral energy), the framework dynamically identifies and emphasizes reliable spectral channels while down-weighting noisy ones. This is particularly critical in PCCT where different energy channels exhibit vastly different photon statistics, low-energy channels suffer from high noise due to photoelectric absorption, while high-energy channels benefit from better penetration but lower contrast. Our automatic weighting strategy ensures that reconstruction leverages the most informative channels without manual parameter tuning, leading to improved spectral fidelity and reduced noise propagation.

Moreover, the hierarchical block clustering with Ward linkage addresses a fundamental weakness of conventional k-means approaches: initialization sensitivity and non-deterministic behavior. In medical imaging, reproducibility is paramount for clinical translation. Our hierarchical strategy builds a complete similarity tree of block relationships, then optimally partitions this tree to minimize within-cluster variance. Unlike k-means, this approach yields identical results across runs, eliminates local minima issues, and naturally discovers material-specific groupings in spectral data. The deterministic nature ensures that identical input data produces identical reconstructions, a critical requirement for quantitative medical imaging and longitudinal studies. Additionally, the variance-adaptive block sizing mechanism accounts for differences in overall structural complexity across images by automatically selecting an appropriate block size based on the global image variance. Images exhibiting higher global variance are processed using smaller 6 × 6 blocks to better capture fine anatomical structures and preserve spatial details, whereas images with lower global variance are processed using larger 8 × 8 blocks to facilitate effective noise suppression in relatively homogeneous images. By selecting a single block size for each image rather than applying a fixed size across all images, this adaptive strategy aims to achieve a better balance between noise reduction and structural preservation, particularly for fine anatomical features such as trabecular bone patterns and tissue boundaries. Integration within the tensor framework exploits the inherent low-rank structure of multi-energy CT data while sparsity-promoting *ℓ*_0_-gradient minimization preserves sharp edges. Comprehensive validation demonstrates the framework’s superiority across sparse-view scenarios. Visual comparisons reveal substantially cleaner images with preserved fine anatomical details that would be lost in competing methods. In addition to the quantitative improvements shown in PSNR, SSIM and FSIM qualitative visual inspection of the reconstructed images indicates that the proposed method better preserves important anatomical structures. Particularly, the cortical bone boundaries in the wrist region appear sharper and more clearly defined compared with the competing reconstruction methods. The internal trabecular patterns within the bone are also more clearly visible, suggesting improved preservation of fine structural details. At the same time, noise levels in soft-tissue regions are reduced, resulting in improved overall image clarity while maintaining structural fidelity. The quantitative results for all five energy channels are summarized in Fig. 7. Across all evaluated energy channels, the proposed STABLE-PCCT framework consistently outperforms the comparison methods in terms of SSIM, PSNR, and FSIM. The performance remains stable across the spectral channels, indicating that the proposed adaptive weighting, deterministic clustering, and variance-adaptive block sizing effectively exploit inter-channel correlations while preserving image quality throughout the entire spectral range. In future work, we plan to further validate the proposed framework using additional datasets acquired from different emerging PCCT systems, extend the evaluation to a wider range of anatomical regions and organs, and investigate its application to downstream spectral imaging tasks such as material decomposition. These studies will help further assess the potential clinical applicability of the proposed method.

In conclusion, we present a novel PCCT reconstruction framework with three synergistic innovations: enhanced multi-metric channel weighting with automatic noise estimation, deterministic hierarchical clustering, and variance-adaptive block sizing. The deterministic approach ensures reproducibility critical for clinical translation. This work advances sparse-view spectral CT imaging while maintaining computational practicality, with extensibility to hybrid learning-based approaches for future clinical deployment.

## Figures and Tables

**FIGURE 1.**
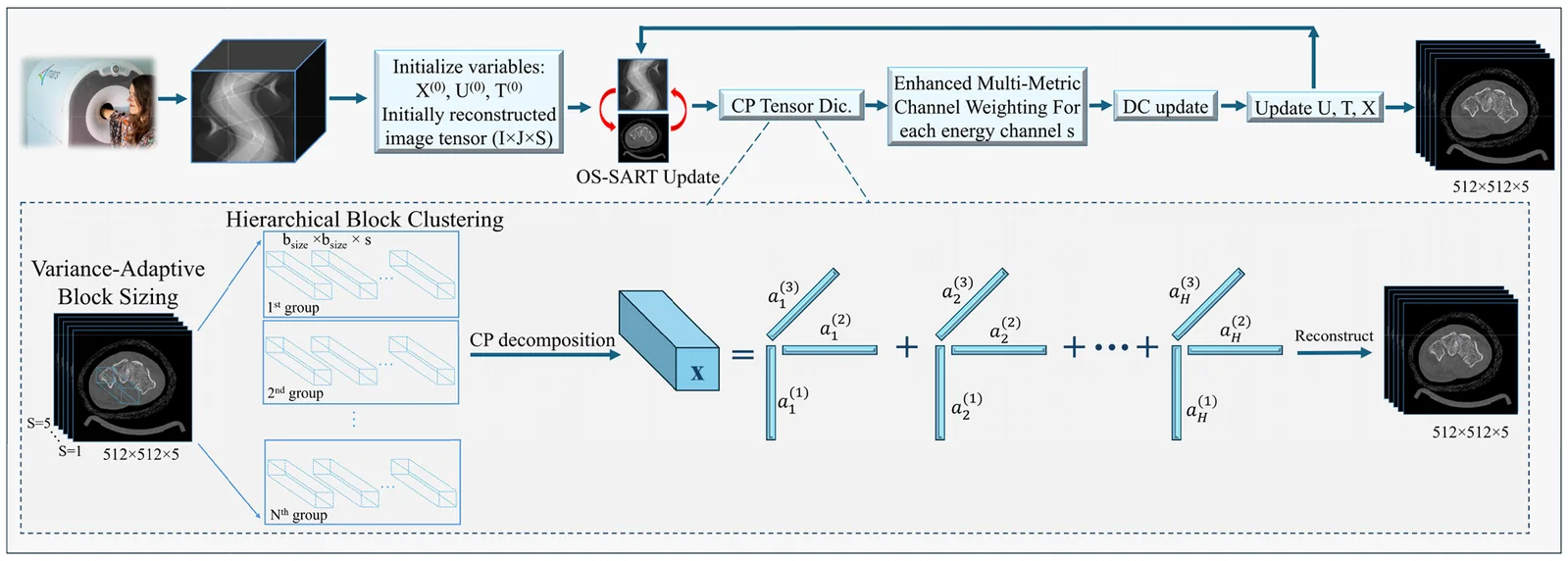
Overall workflow of the proposed PCCT reconstruction framework. The pipeline illustrates the sequential processing steps of the proposed method. It begins with initialization of the image tensor, followed by iterative OS-SART reconstruction integrated with a CP-tensor prior, enhanced multi-metric channel weighting, and data-consistency (DC) updates that enforce agreement between the reconstructed image and the measured projection data. The reconstructed spectral image tensor (X) is iteratively refined to produce a final spectral volume of size 512 × 512 × 5. The magnified block at the bottom illustrates the detailed processing steps, including variance-adaptive block sizing, deterministic hierarchical block clustering, CP tensor decomposition, and block aggregation for collaborative nonlocal denoising while preserving spatial–spectral fidelity. The auxiliary tensors U and T are introduced in the split-Bregman optimization framework to facilitate the *ℓ*_0_-gradient regularization and iterative reconstruction updates, respectively.

**FIGURE 2.**
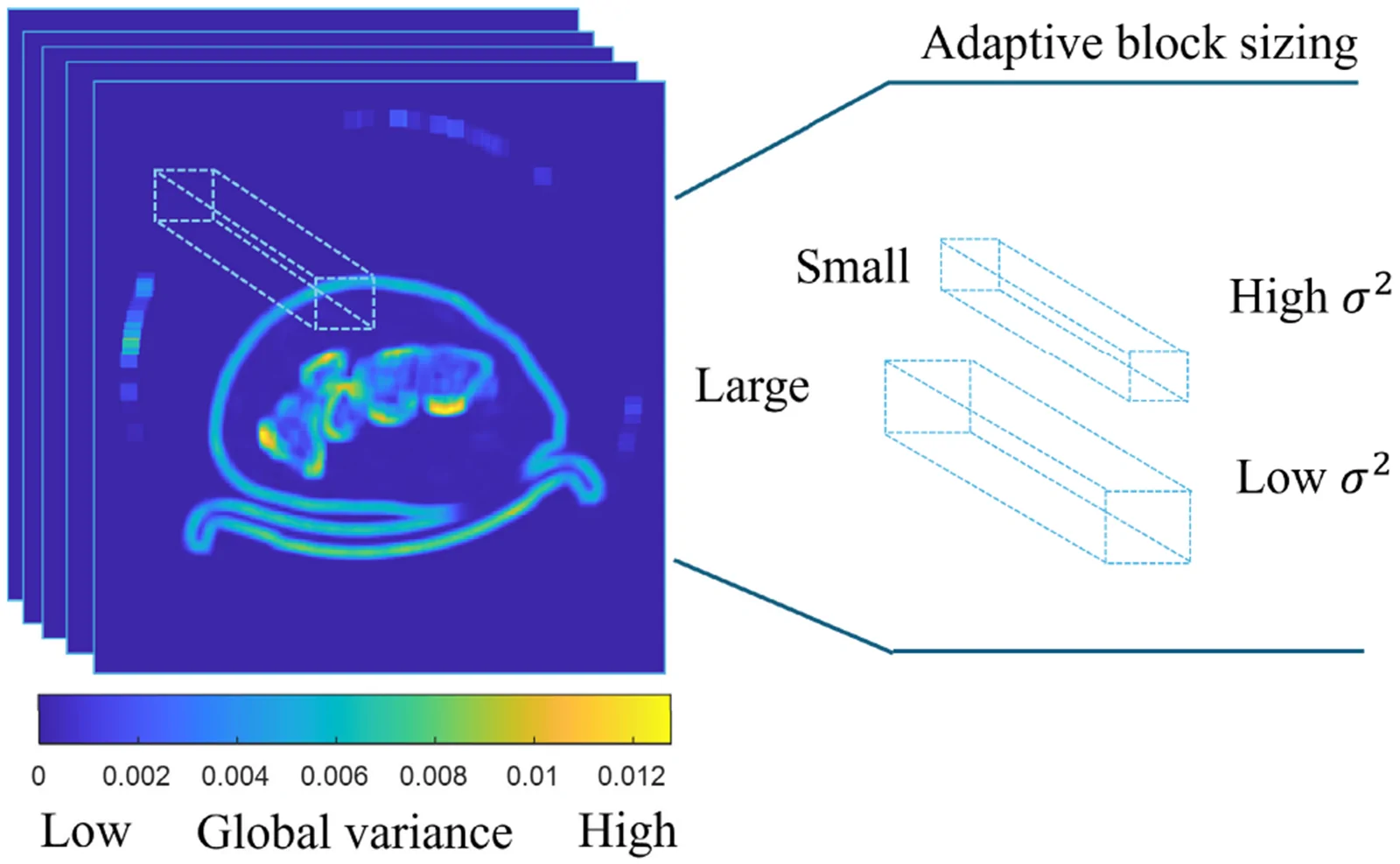
Variance-adaptive block sizing: computing global variance *σ*^2^ of $X\in R^{I\times J\times S}$ to determine a single block size for the entire image. Images with higher global variance use 6×6 blocks, whereas images with lower global variance use 8×8 blocks.

**FIGURE 3.**
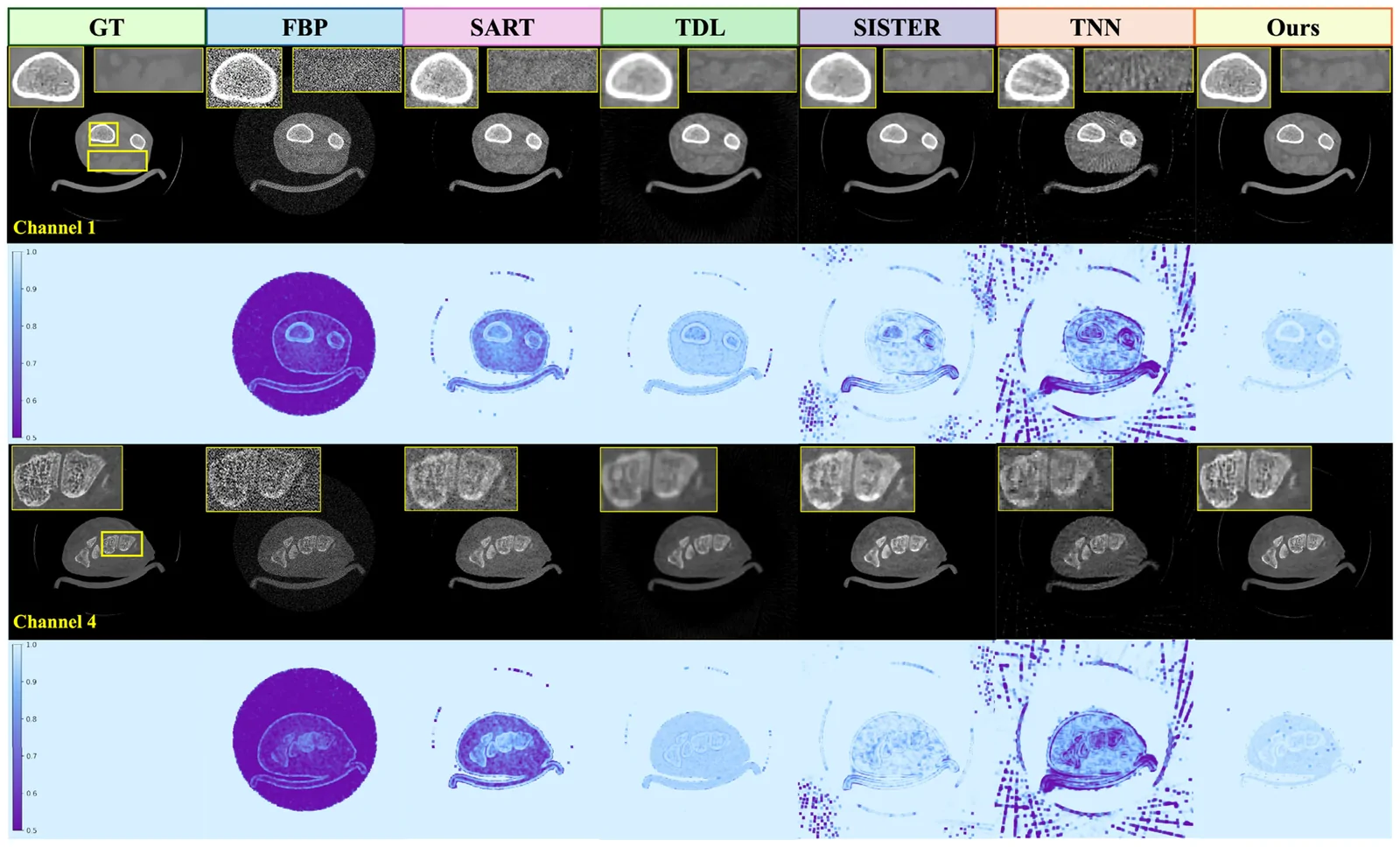
Representative images with magnified ROIs. They are reconstructed for 1^*st*^ and 4^*th*^ energy channels from sparse-view (150 projections) sinograms. The display window is [0, 0.5] mm^−1^ for the first and third rows. The second and forth rows are the SSIM maps between each reconstruction and the reference image, illustrating local structural similarity. Images are 512 × 512 pixels in size.

**FIGURE 4.**
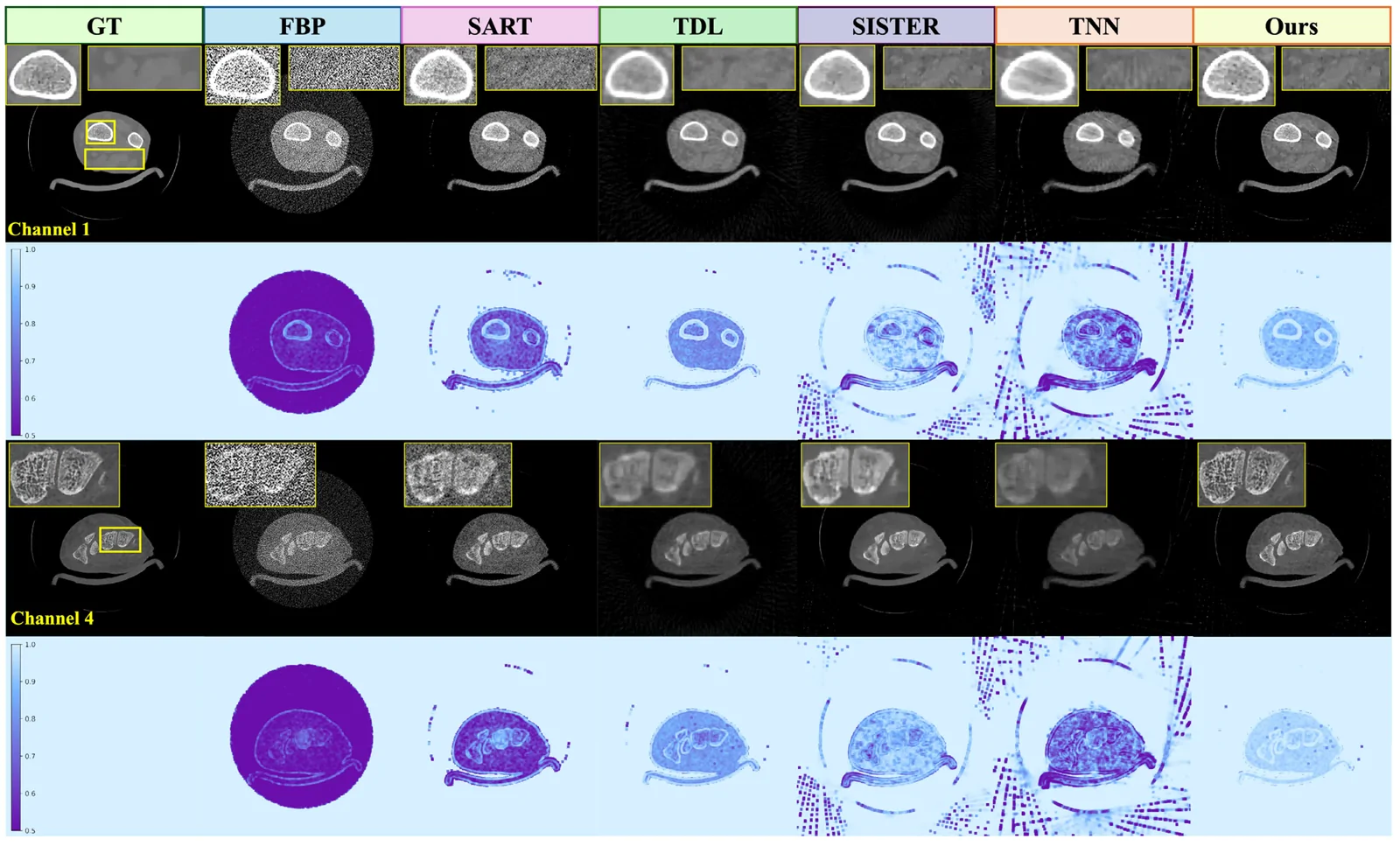
Same as Fig. 3 but from 100 projections.

**FIGURE 5.**
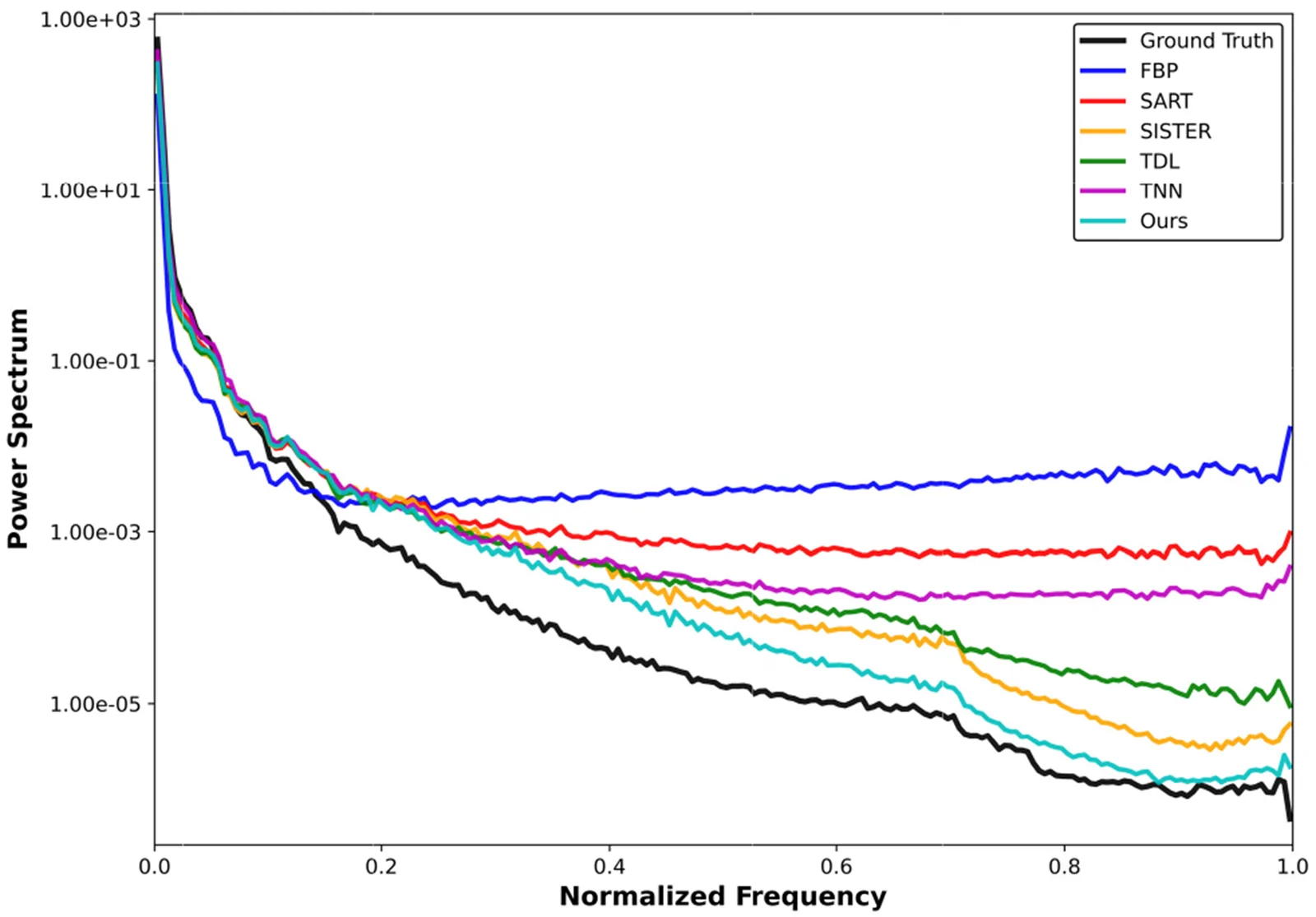
Power spectrum comparison of different reconstruction methods under the sparse-view scenario (150 projections). The horizontal axis represents the normalized spatial frequency, and the vertical axis shows the corresponding power spectrum on a logarithmic scale. A curve closer to the ground truth spectrum indicates better preservation of the image frequency characteristics. The proposed method shows the closest agreement with the ground truth across a wide frequency range, demonstrating improved preservation of structural information while effectively suppressing high-frequency noise.

**FIGURE 6.**
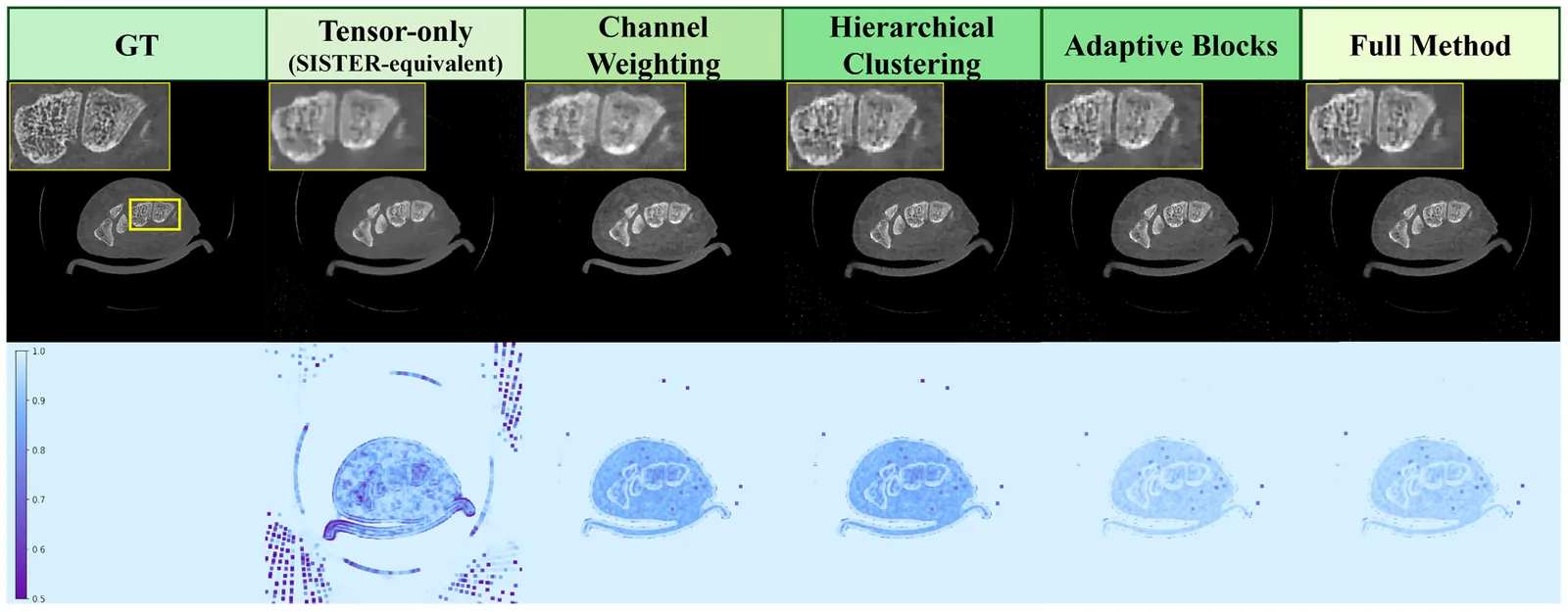
Ablation study results with magnified ROIs evaluated on MARS PCCT (100 projections) for the 4^*th*^ energy channel. The display window is [0, 0.48] mm^−1^. The bottom row shows the SSIM maps between each reconstruction and the reference image. Images are 512 × 512 pixels in size.

**FIGURE 7.**
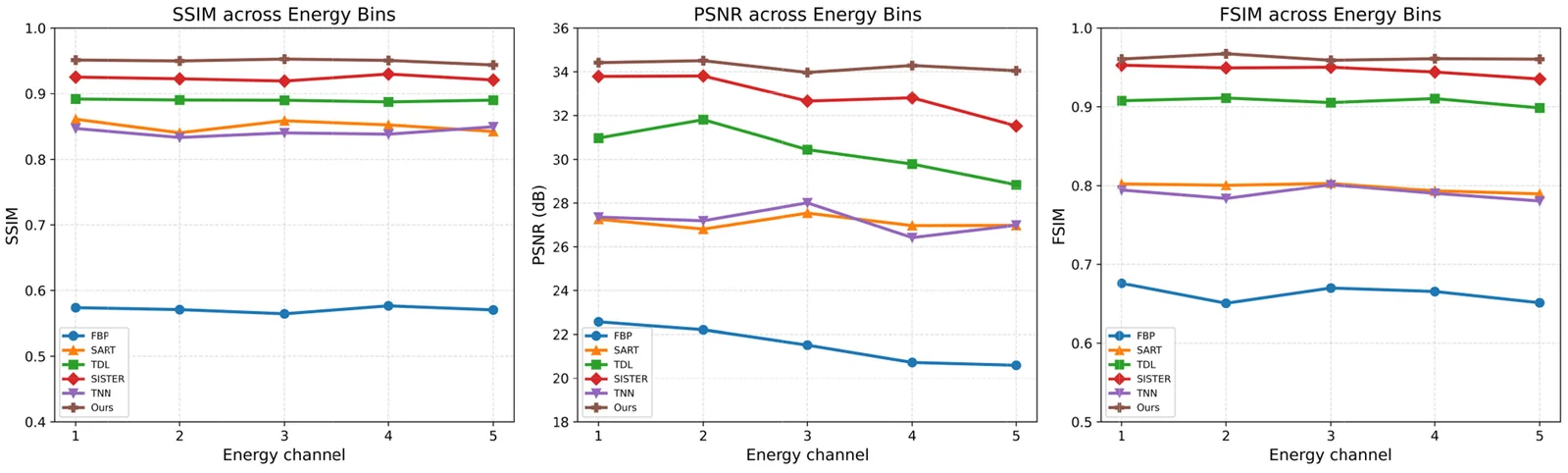
Performance comparison of different reconstruction methods in terms of SSIM, PSNR, and FSIM across the five energy channels for real MARS PCCT data using 100 projection views. The proposed STABLE-PCCT framework consistently achieves superior quantitative performance across all spectral channels, demonstrating stable and robust reconstruction quality under sparse-view acquisition.

**TABLE 1.** List of abbreviations and acronyms used in the paper.

Abbreviation	Description
PCCT	Photon-counting computed tomography
CT	Computed tomography
PCD	Photon-counting detector
SNR	Signal-to-noise ratio
FBP	Filtered backprojection
PRISM	Prior Rank, Intensity, and Sparsity Model
MCT	Multi-energy CT
TDL	Tensor dictionary learning
CNN	Convolutional neural network
GAN	Generative adversarial network
SISTER	Spectral-Image Similarity-based Tensor with Enhanced-Sparsity Reconstruction
MAD	Median absolute deviation
DC	Data-consistency
CP	Candecomp/Parafac tensor decomposition
SSIM	Structural Similarity Index Measurement
PSNR	Peak Signal-to-Noise Ratio
FSIM	Feature Similarity Index Measure
SART	Simultaneous algebraic reconstruction technique
TNN	Tensor neural network

**TABLE 2.** Dataset characteristics and evaluation protocol.

Category	Details
Dataset Source	Clinical extremity scans (wrist), MARS PCCT scanner (MARS Bioimaging Ltd., New Zealand)
Detector Configuration	12 chips arranged in 3 quads (4 chips per quad), forming an arc-shaped camera
Pixel Size	0.11 × 0.11 mm^2^
Detector Grid	128 × 128 pixels per chip
Number of Views	373 views per rotation
Energy Range	7–70 keV
Spectral Channels	5 channels
Source-to-Detector Distance	271.88 mm
Source-to-Object Distance	199.52 mm
Reconstructed Image Size	512 × 512 pixels per slice
Data Type	Photon-counting spectral projection data
Preprocessing	Helical cone-beam to fan-beam rebinning
Evaluation Method	Comparison with full-view reference reconstruction
Quantitative Metrics	PSNR, SSIM, FSIM
Testing Scenarios	Sparse-view configurations *(e.g.,* 150 and 100 views)
Ethical Approval	UMass Lowell Ethics Committee (No. 23-054)

**TABLE 3.** Quantitative evaluation results for the sparse-view scenario (150 projections) using different methods with respect to the reference images. The best results are marked in bold.

Method	SSIM ↑		PSNR (dB) ↑		FSIM ↑	

	1*^st^* Channel	4*^th^* Channel	1*^st^* Channel	4*^th^* Channel	1*^st^* Channel	4*^th^* Channel
FBP	0.5889±0.0448	0.5846±0.0399	23.76±0.1287	21.69±0.0966	0.6897±0.0236	0.6985±0.0426
SART	0.8716±0.0273	0.8551±0.0193	28.64±0.0284	26.76±0.0351	0.8197±0.0374	0.8019±0.0199
TDL [47]	0.9091±0.0109	0.9094±0.0149	32.93±0.1187	31.55±0.0764	0.9112±0.0276	0.9193±0.0266
SISTER [43]	0.9353±0.0242	0.9489±0.0181	34.79±0.1295	35.81 ±0.0462	0.9527±0.0176	0.9589±0.1078
TNN [14]	0.8668±0.0187	0.8482±0.0227	28.56±0.2673	27.57±1.1003	0.8078±0.1208	0.7968±0.0967
Ours	**0.9599±0.0174**	**0.9640±0.0198**	**36.15±0.1257**	**36.35±0.0693**	**0.9675±0.1176**	**0.9698±0.1043**

**TABLE 4.** Quantitative evaluation results for the sparse-view scenario (100 projections) using different methods with respect to the reference images. The best results are marked in bold.

Method	SSIM ↑		PSNR (dB) ↑		FSIM ↑	

	1*^st^* Channel	4*^th^* Channel	1*^st^* Channel	4*^th^* Channel	1*^st^* Channel	4*^th^* Channel
FBP	0.5738±0.0398	0.5766±0.0302	22.57±0.0464	20.72±0.1096	0.6759±0.0659	0.6654±0.0379
SART	0.8610±0.0301	0.8523±0.0266	27.26±0.1483	25.87±0.0389	0.8023±0.0708	0.7933±0.0851
TDL [47]	0.8918±0.0124	0.8874±0.0243	30.97±0.0688	29.79±0.1291	0.9076±0.0519	0.9104±0.0927
SISTER [43]	0.9253±0.0133	0.9299±0.0098	33.79±0.0074	32.81±0.0081	0.9529±0.0208	0.9442±0.0364
TNN [14]	0.8469±0.0049	0.8382±0.0121	27.36±0.0664	26.42±0.0155	0.7943±0.0323	0.7901±0.0449
Ours	**0.9513±0.0101**	**0.9507±0.0162**	**34.42±0.0152**	**34.29±0.0099**	**0.9607±0.0098**	**0.9611±0.0758**

**TABLE 5.** Sensitivity analysis of the threshold parameter *τ*. Reconstruction performance of the proposed method for different values of *τ*, demonstrating that *τ* = 0.20 provides the best trade-off between detail preservation and noise suppression on the MARS PCCT dataset (100 projections) for energy channel 1. The best results are marked in bold.

Threshold *τ*	SSIM↑	PSNR (dB)↑	FSIM↑
0.10	0.9452±0.0104	34.08±0.0047	0.9534±0.0011
0.15	0.9486±0.0098	34.27±0.0350	0.9575±0.0077
**0.20**	**0.9513±0.0101**	**34.42±0.0152**	**0.9607±0.0098**
0.25	0.9494±0.0107	34.21±0.0555	0.9514±0.0009
0.30	0.9468±0.0112	33.98±0.0059	0.9496±0.0049

**TABLE 6.** Sensitivity analysis of the weighting coefficients *α*, *β* and *γ* for the proposed enhanced multi-metric channel weighting strategy on the MARS PCCT dataset for energy channel 1 (100 projections). The best results are marked in bold.

*α*	*β*	*γ*	SSIM↑	PSNR (dB)↑	FSIM↑
0.3	0.5	0.2	0.9475±0.0030	34.16±0.020	0.9581±0.0024
0.4	0.4	0.2	0.9492±0.0027	34.31±0.018	0.9601±0.0022
**0.5**	**0.3**	**0.2**	**0.9513±0.0101**	**34.42±0.0152**	**0.9607±0.0098**
0.6	0.2	0.2	0.9497±0.0028	34.23±0.022	0.9578±0.0023
0.5	0.2	0.3	0.9489±0.0031	34.18±0.021	0.9595±0.0025

**TABLE 7.** Ablation study (MARS PCCT, 100 projections) for the 1^*st*^ and 4^*th*^ energy channels (Ch). The best results are marked in bold.

Method	SSIM ↑		PSNR (dB) ↑		FSIM ↑	

	Ch1	Ch4	Ch1	Ch4	Ch1	Ch4
Tensor-only (SISTER-eq.)	0.9253±0.0133	0.9299±0.0098	33.79±0.0074	32.81±0.0081	0.9529±0.0208	0.9442±0.0364
Channel Weighting	0.9389±0.0011	0.9351±0.0025	33.91±0.003	33.11±0.0023	0.9559±0.0024	0.9506±0.0069
Hierarchical Clustering	0.9422±0.0118	0.9378±0.0037	33.98±0.0015	33.57±0.0062	0.9568±0.0108	0.9537±0.0064
Adaptive Blocks	0.9412±0.0103	0.9419±0.0061	34.09±0.0111	33.96±0.0034	0.9584±0.0048	0.9589±0.0064
Full Method	**0.9513±0.0101**	**0.9507±0.0162**	**34.42±0.0152**	**34.29±0.0099**	**0.9607±0.0098**	**0.9611 ±0.0758**

**TABLE 8.** Computational cost and memory usage of representative tensor-based spectral CT reconstruction methods under the same experimental setting. All methods are reproduced and evaluated using 150 projection views. Runtime corresponds to the complete image reconstruction.

Method	Runtime (min)	Memory (MB)
TDL [47]	43.6	396
TNN [14]	6.8	550
SISTER [43]	168.0	643
**Proposed**	**140.0**	**654**
